# Mites of sheep and goats in Oromia Zone of Amhara Region, North Eastern Ethiopia: species, prevalence and farmers awareness

**DOI:** 10.1186/s12917-015-0433-6

**Published:** 2015-05-24

**Authors:** Ahmed Yasine, Bersissa Kumsa, Yacob Hailu, Dinka Ayana

**Affiliations:** Wollo University, School of Veterinary Medicine, Pathology and Parasitology Team, P.O. Box 1145, Dessie, Ethiopia; Department of Parasitology, College of Veterinary Medicine and Agriculture, Addis Ababa University, P.O. Box 34, Bishoftu, Ethiopia; Aix Marseille Université, URMITE, UM63, CNRS 7278, IRD 198, Inserm 1095, Marseille, 13005 France

**Keywords:** Mites, Oromia zone, Prevalence, Goats, Sheep

## Abstract

**Background:**

Mites are one of the most common and widely distributed ectoparasites of small ruminants in Ethiopia, contributing to major hindrances in livestock productivity in the country. Despite of this fact, specific study was not conducted on mites of small ruminants in Ethiopia. Therefore, the present study was performed from October 2009 to May 2010 to determine the prevalence and species composition of mites in three agroecological zones in north eastern Ethiopia. In addition, a questionnaire survey on mites was conducted to assess the control practices and awareness of farmers in the study areas.

**Results:**

Out of a total of 1280 sheep and 1264 goats examined, 97(7.6 %) of sheep and 174(13.8 %) goats were infested with one or more species of mites. In goats an overall prevalence of 10.3 % *Sarcoptes*, 2.8 % *Demodex* and 0.6 % *Psoroptes* were recorded whereas in sheep an overall prevalence of 3.5 % *Sarcoptes*, 2.1 % *Demodex* and 1.6 % *Psoroptes* were observed. *Sarcoptes* (*P* = 0.03; OR = 2.1) and *Demodex* (OR = 3.25; *p* = 0.004) were significantly more common in young than in adult sheep. Demodectic mange was significantly higher in young (4.1 %) compared to adult (2.3 %) goats (OR = 2.2; *P* = 0.02). Significantly higher (*P* < 0.01) overall prevalence of sarcoptic and demodectic mites in both sheep and goats with poor than with good body condition was recorded. Results of the questionnaire survey supported results of our cross-sectional study.

**Conclusions:**

This study demonstrates high prevalence of mange mites in sheep and goats of the study area. The study revealed that S*arcoptes* is the predominant mite in both sheep and goats. Animal owners and veterinarians should consider mite control in small ruminants as part of the routine ectoparasite control in Ethiopia.

## Background

Ectoparasites such as mites, ticks, lice and fleas affect large number of sheep and goats in Ethiopia [1, 2]. They are one of the major hindrances to the productivity of small ruminants in the country. Ectoparasites cause a wide range of health problems including mechanical tissue damage, irritation, inflammation, hypersensitivity, abscesses and predispose to myiasis and dermatophilosis. Infestations increase susceptibility to other diseases and create sites for secondary invasion by pathogenic organisms and reduced productivity [3]. Furthermore, the damage caused by mites, ticks, lice, and fleas are responsible for the downgrading and rejection of sheep and goat skins. In Ethiopia 35 % of sheep and 56 % of goat skin rejections are attributed to ectoparasites [4].

Mange is a highly contagious, widespread condition that can be transmitted between animals by direct and indirect contact [4]. The economic impact of mites in infested animals comes from retarded growth, reduced daily weight gain, cost of treatments and labour, damage to the skin and hides and mortalities [5]. In addition, mites severely reduce the well-being of animals, reducing milk yield and hamper the milking process due to the restlessness of affected animals. Moreover, some mite species have zoonotic and public health importance [6, 7]. The export of processed and semi-processed skins earns the second largest income next to coffee export in Ethiopia. However, several recent reports indicate that over the last decade the quality of skins of small ruminants has deteriorated from the effects of increase in the prevalence of infestation with parasitic arthropods [1, 2, 8]. It is commonly believed that mites played an important role for this continuous declining in quality of skin of small ruminants. Despite all these facts, to the best of our knowledge all the previous reports on mites of sheep and goats in Ethiopia resulted from studies of other ectoparasites, specifically ticks and lice. Therefore, the present study was conducted to determine the prevalence, species and risk factors associated with mange mites of sheep and goats in Oromia zone of Amhara regional state in north eastern Ethiopia. In addition, a questionnaire survey was used to determine the control practices and awareness of farmers about mites of sheep and goats in the study areas.

## Methods

### Study area and animals

The study was conducted in Oromia zone which is located in Amhara Regional state in north eastern Ethiopia. It is located at 325 km North of Addis Ababa. The geographical location of the area is between 10^0^ 01′ N to 11° 25′ N and 39° 41′E to 40° 24′ E. The altitude of the area ranges from 1000 to 2500 m above sea level and the maximum temperature is 33 °C and the minimum temperature is about 12 °C. The mean annual rainfall of the area ranges from 600 mm to 900 mm. The area receives long heavy rainy season from June to September and short rainy season from March to May [9]. The area comprises various soil types including clay soil (18.8 %), sand soil (44.6 %) and loam soil (36.6 %). The estimated animal population of the area is 266,000 cattle, 200,000 sheep and goats, 21,000 equines, 7400 camels and 151,000 chickens. Out of the total of 392,684 ha of land area of the zone, 15 % is used for crop production, 25.3 % for animal grazing, 4.96 % forest covered and 37.4 % bush covered [9].

Indigenous sheep and goats found in Dawacheffa and Bati districts of Oromia zone in Amhara Regional State were studied for mange mites from October 2009 to May 2010. In Dawacheffa district mixed crop-livestock is the common type of animal production system whereas agro-pastoral type is practiced in Bati district. All the animals are kept under extensive management system in the different agro-climates. The agroecology of the study area was categorized into highlands (>2000 m), midlands (1500–2000) and lowlands (<1500 m).

### Study design

#### Questionnaire survey

Questionnaire survey was conducted to obtain information on the magnitude of mites of small ruminants, assess the awareness and control practices against mange mites and evaluate risk factors on the occurrence of the disease associated with mites. Five sheep and goat owners were selected for interview from six peasant association (PA) purposively based on accessibility making a total of 90 individuals (30 farmers from each of the three agroecological zones).

#### Cross sectional survey

Six PAs from each agroecological zones were identified. Sheep and goats in the PAs brought for routine vaccination programs were randomly selected. The sample size for the study was determined as described by Thrusfield [10] taking an estimated prevalence of 33.27 % of mange mites [11]; accepted error 5 % and confidence level of 95 %. Thus, for the present study 420 sheep and 420 goats from each agroecological zones was the minimum sample size. But to increase the statistical accuracy a total of 2544 animals (1280 sheep and1264 goats) were examined to determine the prevalence, risk factors and species of mange mites in the study areas.

#### Body condition score determination

A total of 2544 randomly selected animals (1280 sheep; 1264 goats) from the three agroecological zones were carefully examined to determine their body condition score. Prior to clinical examination the origin, age and sex of each selected animal was recorded. Body condition score was determined by modifying the scoring system described by Gatenby [12] and Steele [13] for sheep and goats, respectively. Poor body condition score was assigned to sheep and goats that were extremely thin and with smooth and prominent spinous process and transverse process and in which finger can be pushed and those with moderate depth of loin muscle. Good body condition score was given to those sheep and goats in which the spinous process only stickup very slightly; smooth, rounded and well covered transverse process and with full loin muscle.

#### Mite collection and identification

From each affected animal mite samples were collected into pre-labelled small plastic tubes from deep skin scraping until capillary oozing was evident. In the laboratory each sample was treated with 10 % potassium hydroxide solution for 10 min and then examined under light microscope. When nodular skin lesions were suspected to be due to demodectic mange, white creamy pus content was collected and subjected to direct smear microscopic examination. Skin scrapings for different species of mange mites and species identification was performed according to taxonomic morphological key characteristics described by Wall and Shearer [6] and Taylor et al. [7].

#### Ethical approval

Ethical approval for the collection of mite samples from goats and sheep was obtained from the animal research ethics board (Agreement # 07/09/2009) of the College of Veterinary Medicine and Agriculture of Addis Ababa University. All necessary permits were obtained from the administration and agricultural office of each district and from each animal owner and from each interviewed person for questionnaire survey. The collection of mite samples in the field did not involve privately owned, wildlife, national park or other protected areas and endangered or protected species.

#### Data analysis

Raw data was carefully recorded and stored in Microsoft Excel database system used for data management. Statistical software package called SPSS for windows version 17.0 was used for data analysis. Descriptive statistics, percentages and 95 % confidence intervals were used to summarize the proportion of infested and non-infested animals. The effects of different environmental and host risk factors were analyzed by regression and *χ*2 test. Statistical significance was set at *p* ≤ 0.05.

## Results

### Questionnaire survey

Results of the questionnaire survey showed that sheep and goats in the study area are kept by farmers mainly for income generation and insurance 74/90 (82.2 %) followed by home meat consumption 16/90 (17.8 %). Majority of the respondents 54.5 % informed that mange mites affect young animals whereas 24.4 % of them replied mange usually affect adult small ruminants. In addition, 81.1 % of them indicated mite is common during the dry season whereas 4.5 % of the respondents replied mange is problem during the wet season (Table 1). Moreover, 70 % of the respondents were aware that mange is a problem in goats however, only 3.3 % of them mentioned mange in sheep (Table 1). 95.5 % of the respondents explained they used modern treatments in veterinary clinics whereas the rest (4.5 %) indicated the use of some traditional treatments. 97.78 % of the respondents indicated the participation of farmers in the control practices against the disease launched by the government (Table 1).

**Table 1:** Summary of the questionnaire survey of mites of small ruminants in the study area

Focal points	Percentage of response (%) (*n* = 90)
Age groups of animals affected	
Adults	24.4
Young	54.4^a^
All age groups	21.1
Seasonality of mange	
Wet	4.5
Dry	81.1^a^
Equally	14.4
Effects of mange mites on	
Sale of live animals	92.2^a^
Sale of skin	77.8
Animal spp more affected by mites	
Sheep	3.3
Goats	70^a^
Equally	25.7
Ways of treatment	
Modern	95.5^a^
Traditional	4.5
Participation of farmers in the control practice	
against the disease launched by the government	
Yes	97.78^a^
No	2.22

^a^show significantly higher percentage

### Overall prevalence of mange mites

Of the total 1280 sheep and 1264 goats examined for mange mites, 97(7.6 %) sheep and 174(13.8 %) goats were infested at least with one species of mange mites (Table 2). *Sarcoptes* was identified as the predominant mites followed by *Demodex* and *Psoroptes* in both sheep and goats. The overall prevalence of mange mites is significantly (*p* < 0.01) higher in goats (13.8 %) than in sheep (7.6 %).

**Table 2:** Overall prevalence of mites in sheep and goats

	Sheep (*n* = 1280)			Goat (*n* = 1264)		
Mite spp.	No. positive	Prevalence (%)	95 % CI	No. positive	Prevalence (%)	95 % CI
*Sarcoptes* spp	49	3.8	2.75–4.84	130	10.3	8.63–11.96
*Demodex* spp	27	2.1	1.31–2.88	36	2.8	1.89–3.70
*Psoroptes* spp	21	1.6	1.21–1.95	8	0.6	0.18–1.02
Overall	97	7.6	6.15–9.05	174	13.8	11.91–15.68

### Prevalence of mange mites by agroecology

The overall prevalence of mange mites in highland, midland and lowland was 2.6 %, 14.2 % and 5.9 % in sheep and 0.9 %, 22.8 % and 17.7 % in goats, respectively (Table 3). The overall prevalence of mange mite infestation is significantly (*p* < 0.01) higher in midland than both lowland and highland agroecological zones. In sheep the overall prevalence of *Sarcoptes* and *Demodex* spp. were significantly higher in the midland and lowland than the highland agroecology. However, the overall prevalence of *Psoroptes* spp. was significantly higher in the highland and midland than the lowland agroecology (Table 3).

**Table 3:** Prevalence (%) of mites in sheep and goats in three agro-ecological zones

	Sheep			Goat		
	High land	Mid land	Low land	High land	Mid land	Low land
Mites spp.	*n* = 425	*n* = 430	*n* = 425	*n* = 424	*n* = 417	*n* = 423
*Sarcoptes*	0	7.9	3.5	0	17.5	13.5
*Demodex*	0	3.9	2.4	0	4.6	4.0
*Psoroptes*	2.6	2.3	0.0	0.9	0.7	0.2
Overall	2.6	14.25	5.9	0.9	22.8	17.7

In goats the effect of agroecology on the prevalence of mange mites was analyzed using logistic regressions. Despite the presence of variations in the prevalence values, statistically significant (*P* > 0.05) differences was not observed among agroecological zones in all the three genera of mange mites infesting goats (Table 3).

### Prevalence of mange mites by age

The overall prevalence of mange mites in young and adult sheep was 10.2 % and 6.8 %, respectively (Table 4). Significantly higher prevalence of *Sarcoptes* spp (OR = 2.1; *P* = 0.03) and) *Demodex* spp (OR = 3.25; *p* = 0.004) in young than adult sheep was noted. The overall prevalence of mange mites in young and adult goats was 13.0 % and 14.1 %, respectively (Table 4). The overall prevalence of demodectic mange was significantly (OR = 2.2; *p* = 0.02) higher in young than adult goats (Table 4).

**Table 4:** Prevalence (%) of mites in sheep and goat according to age group

	Sheep^a^				Goats^b^			
	Adult (*n* = 976)		Young (*n* = 304)		Adult (*n* = 902)		Young (*n* = 362)	
Mites spp.	No. positive	Prevalence (%)	No. positive	Prevalence (%)	No. positive	Prevalence (%)	No. positive	Prevalence (%)
Sarcoptes*	32	3.3	17	5.6	99	10.9	31	8.6
Demodex**	14	1.4	13	4.3	21	2.3	15	4.1
Psoroptes***	20	2.0	1	0.3	7	0.8	1	0.3
Overall	65	6.8	31	10.2	127	14.1	47	13.0

^a^ *OR = 2.1 *p* =0.034 0.05; ^b^ *O R = 0.8 *p* =0.5

**OR = 3.25 *P=*0.004; **OR = 2.2 *P=*0.02

***OR = 0.163 *P* = 0.07; ***OR = 0.33 *P*= 0.31

### Prevalence of mange mites by body condition score

An overall prevalence of 4.7 % and 41.1 % mites in sheep and 7.2 % and 55.6 % in goats was recorded in animals with good and poor body conditions, respectively (Table 5). In both sheep and goats, significantly (*p* < 0.01) higher overall prevalence of mange mites was recorded in animals with poor than those with good body condition. Significantly higher prevalence of *Sarcoptes* spp. (OR = 22.32; *P* < 0.01), *Demodex* spp. (OR = 7.8; *p* < 0.01) and *Psoroptes* spp. (OR = 4.7; *p* = 0.01) in sheep with poor than good body condition scores was recorded. In goats significantly higher prevalence of sarcoptic (*p* < 0.01; OR = 27.1) and demodectic mange (*p* < 0.01; OR = 5.1) was associated with poor than good body condition scores.

**Table 5:** Prevalence of mites in sheep and goat according to body condition

	Sheep				Goats			
	Good (*n* = 1181)		Poor (*n* = 99)		Good (*n* = 1093)		Poor (*n* = 171)	
Mites spp	No. positive	Prevalence (%)	No. positive	Prevalence (%)	No. positive	Prevalence (%)	No. positive	Prevalence (%)
*Sarcoptes*	24	2.0	25	25.3	53	4.8	77	45.0
*Demodex*	18	1.5	9	9.1	21	1.9	15	8.8
*Psoroptes*	14	1.2	7	7.1	5	0.5	3	1.8
Overall	56	4.7	41	41.1	79	7.2	95	55.6

## Discussion

The present study demonstrated that mange mites are one of the most important ectoparasites of small ruminants of all age groups, both sexes and body conditions in all agroecological zones of the study area. Poor health management, malnutrition and lack of good knowledge about mange mites of animal owners have been suggested as favorable factors for this widespread occurrence of infestation [1, 2]. These facts were supported by the results of the questionnaire survey as owners were usually aware the effects of mites only at the advanced stage of the disease and when irreversible permanent damage is evident. This observation is in line with earlier report in Amhara regional state [8].

The overall prevalence 7.6 % and 13.8 % of mites in sheep and goats, respectively recorded in the present study is higher than the previous prevalence of 2.1 % in sheep and 4.3 % in goats in Sidama zone [14], 0.4 % in sheep and 6.6 % in goats in Amhara Regional State [8], 0 % in sheep and 0.98 % in goats in Wolayta Sodo [15], 1.2 % in sheep in central Oromia [2] and 8.8 % in goats in central Oromia [1]. A possible explanation for this differences in the prevalence among different studies could be variations in environmental and host factors, study seasons, owners knowledge of mites and animal husbandry and managements as has been argued previously [2]. It also implies that the climatic conditions of the current study areas are more suitable for survival, reproduction and development of various stages of mites.

The finding of significantly higher overall prevalence of mite infestation in goats than in sheep in the current study supports earlier studies in Ethiopia [1, 2, 8, 14], in Nigeria [16] and in Iran [17]. The most probable explanation for this is the higher susceptibility of goats to mange mites, and the development of an effective immunity in sheep [16].

The higher overall prevalence of mange mites in the midland and lowland than the highland agroecology in the present study is in agreement with previous reports [2, 8]. This is most probably attributed to the higher temperature, humidity and sunlight prevailing in the midland and lowland agroecological zones suitable for reproduction and multiplication of mites [18]. For similar reasons higher overall prevalence of sarcoptic and demodectic mange infestations in the lowland and midland than in the highland agroecology was recorded. This finding also supports the result of the questionnaire survey that the owners generally noticed mange problem in the midland and lowland than in the highland agroecology especially in goats.

The higher prevalence of sarcoptic and demodecticmites in young than adult sheep and demodectic mange in young than adult goats in the current study is in line with the previous observations [1] and most probably reflects the under-developed immunity in young animals.

The higher prevalence of mites in sheep and goats with poor body condition than those with good body condition in our study supports earlier reports [8, 18]. This finding most probably reflects the higher mite burden in animals with poor body condition that cause decline in live weight and overall productivity as has been argued before [5]. Poor body condition is usually associated with undernourishment which decline immunity increasing susceptibility to pathogens including ectoparasites [7, 19]. On the other hand, well-fed animals in good body condition can withstand infestation challenges.

## Conclusions

This study demonstrated the occurrence and high prevalence of different species of mange mites in sheep and goats of all age groups, both sexes and body conditions in all agroecological zones of the study area. The study revealed that *Sarcoptes* is the predominant mite both in sheep and goats followed by *Demodex* and *Psoroptes* species. Further epidemiological studies on the economic and zoonotic importance of mites in different species of animals, various agroecological zones, breed and management systems warrant urgent attention. Animal owners and veterinarians in Ethiopia should consider mite control in small ruminants as part of routine control of ectoparasites. Appropriate extension programs should be launched to create public awareness about the economic importance, treatments and its impact on skin quality.
